# Automatic PET-CT Image Registration Method Based on Mutual Information and Genetic Algorithms

**DOI:** 10.1100/2012/567067

**Published:** 2012-04-19

**Authors:** Martina Marinelli, Vincenzo Positano, Francesco Tucci, Danilo Neglia, Luigi Landini

**Affiliations:** ^1^Institute of Clinical Physiology, CNR, Via Moruzzi n.1, 56124 Pisa, Italy; ^2^Fondazione Gabriele Monasterio, CNR-Regione Toscana, Via Moruzzi, 1, 56124 Pisa, Italy; ^3^Department of Information Engineering, University of Pisa, Via Diotisalvi, 2, 56126 Pisa, Italy

## Abstract

Hybrid PET/CT scanners can simultaneously visualize coronary artery disease as revealed by computed tomography (CT) and myocardial perfusion as measured by positron emission tomography (PET). Manual registration is usually required in clinical practice to compensate spatial mismatch between datasets. In this paper, we present a registration algorithm that is able to automatically align PET/CT cardiac images. The algorithm bases on mutual information (MI) as registration metric and on genetic algorithm as optimization method. A multiresolution approach was used to optimize the processing time. The algorithm was tested on computerized models of volumetric PET/CT cardiac data and on real PET/CT datasets. The proposed automatic registration algorithm smoothes the pattern of the MI and allows it to reach the global maximum of the similarity function. The implemented method also allows the definition of the correct spatial transformation that matches both synthetic and real PET and CT volumetric datasets.

## 1. Introduction

Cardiac images acquired via different modalities can provide complementary information. Therefore, the fusion of two or more coregistered multimodal datasets into a single representation can provide important support for a medical diagnosis or for the therapeutic evaluation in cardiology. However, the use of a multimodal imaging approach in clinical practice is limited by drawbacks in accurate image alignment. Several approaches have been developed to register 3D cardiac datasets [1]. These approaches can be classified by considering three key components: the domain where the alignment transformation is defined (search space), the function that describes the quality of the alignment (similarity metric or registration metric), and the optimization strategy used to calculate the transformation that maximizes the defined similarity function. For the search space, the rigid transformation method is the easiest to be implemented since only six parameters (three translational and three rotational) are considered. Unfortunately, this approach does not allow a full description of the complex, nonrigid motion of the heart during the cardiac cycle. Elastic registration should be employed to take this into account, but its clinical application is limited by the computational cost needed to perform the registration [2]. The choice of a similarity metric, which indicates goodness of the match, is a key issue in developing a registration method. Indeed, the similarity metric must be robust, such that it converges to a global maximum for a correct matching, and must also be computed in a reasonable time. Different methods use the distance between selected geometrical image features, such as anatomical landmarks [3], surfaces on the thorax [4, 5], or heart surfaces [6, 7], and require definition of the corresponding anatomical points or segmentation of the surfaces. These procedures are not always easy to perform because of the dissimilar information provided by different modalities, especially when a defect in the myocardial wall is present. For these reasons, voxel-based similarity metrics such as the use of mutual information [8–10] are established techniques among multimodality registration metrics because they do not make assumptions about the relationship between given image intensities. The last important component of a registration process is the optimization algorithm, or search strategy, which can calculate the transformation that maximizes the defined similarity metric and aligns the cardiac datasets. Local approaches, such as the Powell [4, 6] or simplex [11, 12] methods, are widely applied since they are rapidly implemented and run. The main drawback of the local approaches is the possibility of convergence to a local maximum, resulting in a nonoptimal value of the transformation matrix. In contrast, global optimization methods such as genetic algorithms (GAs) [13] can achieve a solution near the optimal transformation value but are associated with a higher computational complexity. Multiresolution methods have been proposed to speed up the optimization process and to reach the global maximum of the similarity metric [14].

 Recently, the clinical need to merge complementary information has been emphasized by the success of the hybrid scanners, which are able to acquire in a single imaging session multimodal data that provide complementary information. The most important examples are the PET/CT devices [15, 16], which have found wide cardiac application because of their ability to define the association between coronary artery disease (CAD), as revealed by computer tomography (CT), and myocardial perfusion, as measured by positron emission tomography (PET). Although single-session acquisition generally minimizes misalignment between the PET and CT datasets, the spatial and temporal mismatch of cardiac datasets in particular are not completely resolved. Thus manual registration with an integrated commercial software is usually performed in clinical practice to obtain optimal alignment [17, 18]. For this reason, an automatic method could be useful in cardiac PET/CT matching and when a hybrid scanner is used. Different approaches exist that can align cardiac PET and CT datasets according to thoracic CT images and transmission PET [4, 19]; other techniques draw on algorithms used to match SPECT and CT [20] images.

In this work, we describe an automatic method to register functional PET and anatomical CT 3D cardiac datasets. The developed registration algorithm is based on a rigid transformation described by three translational and three rotational parameters. The mutual information (MI) measure has been used as the similarity metric, and a multiresolution optimization algorithm based on existing genetic algorithms has been implemented that defines the optimal transformation that makes maximum use of the MI value. One real PET and CT dataset has been used to build a registered synthetic model exploited for the development of the registration algorithm and for the evaluation of the similarity metric function and of the optimization approach. The registration algorithm presented here has also been applied to real PET and CT datasets.

## 2. Materials and Methods

### 2.1. Base of Knowledge and Validation Data

Three CT-PET datasets were acquired with PET/CT Discovery-RX (GE Healthcare) from patients scheduled for simultaneous assessment of myocardial perfusion and coronary artery disease. The first dataset was used as the reference when developing the registration algorithm, while the other datasets were used to test the algorithm. The CT datasets were acquired during ECG gating and end-expiratory breath holds and consisted of 411 transaxial slices of 512 × 512 pixels, with voxel size 0.43 mm × 0.43 mm × 0.43 mm. The end-diastolic image volumes were used in the registration process. Static PET datasets were acquired after injection of the radioisotope NH_3 _and reconstructed as 47 transaxial slices of 128 × 128 pixels, with voxel size 3.24 mm × 3.24 mm × 3.24 mm. Informed consent was obtained for all subjects involved. The local institutional review board approved the study.

### 2.2. Hardware and Software Specification

The registration algorithm has been developed in MATLAB 7.0 and the genetic algorithm has been implemented by using the Genetic algorithm and Direct Search Toolbox. The development and the evaluation of the registration algorithm has been done by using an Intel Core i3-530-2.93 GHz 4 MB cache and Windows XP OS.

### 2.3. Synthetic Models

Two isotropic 3D cardiac models were built to simulate registered PET and CT short axis image volumes. Both models were developed using an elliptical geometry and a previously described image model [21].

The CT model was described by the following equation:

(1) $$I\left(\overset{®}{x}\right)=\left\{I_{0}\left(\overset{®}{x}\right)⊗h\left(\overset{®}{x}\right)\right\}+n\left(\overset{®}{x}\right),$$

where $\overset{®}{x}$ represents the voxel position. The ideal image *I*_0_ consisted of piecewise constant regions, with the gray values of the various structures defined by the Hounsfield units that characterize real tissues. The Gaussian convolution kernel *h*  (SD = 0.8) was used to model the partial volume effect of the real images. The last component *n* defined the zero-mean Gaussian-distributed CT noise [22], with a standard deviation corresponding to that measured in a uniform background region.

The PET model was described by the following equation:

(2) $$I_{i}\left(\overset{®}{x}\right)=\left\{I_{0i}\left(\overset{®}{x}\right)⊗h\left(\overset{®}{x}\right)\right\}g\left(\overset{®}{x}\right)+n\left(\overset{®}{x}\right).$$

In comparison to the CT model, the PET model contained only the gray value *I*_0_ of the myocardial wall structure, deducted after observation of real PET images. A larger Gaussian convolution kernel *h*  (SD = 2.4) was applied to take into account the larger partial volume effect in the real PET dataset. Furthermore, a multiplicative Gaussian smooth field $g\left(\overset{®}{x}\right)$ described by

(3) $$g\left(\overset{®}{x}\right)=1-\left(1-a_{\max}\right)\cdot \exp\left(-\frac{\sum_{i}{\left(\overset{®}{x_{i}}-{\overset{®}{x}}_{i_{c}}\right)}^{2}}{2\sigma^{2}}\right)$$

was employed to model the lack of PET signal intensity caused by a pathological condition as perfusion deficit. The term *a*_max⁡_ represents the maximum fading of the intensity signal at position $\overset{®}{x{}_{c}}$ and the parameter *σ* describes the standard deviation of the Gaussian field.

As for the CT model, the PET noise, described by the last term *n*, was modeled with a zero-mean Gaussian distribution with a standard deviation corresponding to that measured in a uniform background region.

### 2.4. Overall Registration Algorithm

Image fusion was based on the 3D registration process used to define the spatial and temporal alignment of the volumetric datasets involved. The aim of the registration process was to define a transformation to map voxels from the reference image volume (*V* ^*r*^) to the floating image volume (*V* ^*f*^):

(4) $$T:V^{f}\to V^{r}\Leftrightarrow T\left(V^{f}\right)=V^{r}.$$

As previously noted, the entire registration process can be described by considering three linked components: the domain where the transformation *T* is defined (search space), the function that defines the quality of the alignment (similarity metric or registration metric), and the optimization strategy used to calculate the transformation *T* that maximizes the defined similarity function. In the next sections, we give a detailed description of each component integrated into the overarching registration algorithm.

#### 2.4.1. Search Space

The transformation definition is based on the geometrical operations required for the process. The 3D rigid transformation has six degrees of freedom (three translational and three rotational parameters), while the affine transformation adds scaling and shearing parameters. The most general transformation is the elastic or nonrigid registration. In theory, it has infinite degrees of freedom and can be represented by a polynomial function. The elastic registration should be used for cardiac image registration, but its clinical application is limited by its high computational load and by the long time needed to perform the registration [2]. In contrast, the rigid transformation is not time-consuming and imposes a low computational load, although it does not allow a full description of the complex nonrigid motion of the heart during the cardiac cycle. This has been confirmed by its application in the currently available commercial clinical software that are most often used to align cardiac PET and CT datasets. For this reason, the rigid transformation has been implemented and used in the automatic registration algorithm presented here.

#### 2.4.2. Similarity Metric

The similarity metric used to evaluate alignment must be robust and must be performed in a reasonable time. In our registration method, the mutual information metric was used because it does not make any assumptions about the relationship between various image intensities [8]. This measure, introduced by Collignon et al. [23] and Viola and Wells [9], is based on the joint histogram calculation and yields the maximum value when the correct spatial alignment is reached. Multiple parameters can affect the MI value and thus the probability of reaching the global and optimal alignment solution. In particular, as previously described [24], a key element in the MI calculation is the choice of interpolation method used to calculate the joint histogram after the geometrical transformation is performed. In fact, when a transformation matrix is applied to the floating image volume, the new voxel intensities at intragrid positions must be estimated by interpolation. Choosing the optimal interpolation method is of fundamental importance because it can modify the gray-level values of the floating dataset, the value of the defined similarity function, and the global convergence of the optimization algorithm. In particular, when the MI is used, it is important to preserve the gray-level distribution and, as extensively described [24], to consider how the interpolator (used to calculate the joint histogram) affects the metric function. Examples of different approaches to interpolation are the nearest neighbor (NN) and the partial volume (PV) methods [10, 25]. The NN method is the simplest interpolation technique because the value of each nongrid voxel is defined using the nearest voxel value. The principal drawback of the NN method is that it is insensitive to intravoxel misalignments. PV interpolation updates the intensities of voxels at nongrid positions using the fractional intensities of their neighbors in grid positions. Trilinear interpolation is used to estimate the fractional weights. As previously described [24], when the PV approach is used, artifacts in the MI appear when the ratio of the spatial resolutions from the two datasets along a given direction is either one or a simple rational fraction. For this reason, a generalization of the PV method, called the generalized partial volume estimation (GPVE), has been introduced by Chen and Varshney [26]. This method is based on varying the reference voxel number involved in joint histogram updating and is accomplished by using different kernel functions. If either a triangular function or first-order B-spline is used, the GPVE method is equivalent to the PV method. Major-order b-spline approaches can be used in order to decrease artifacts. Moreover, different kernel functions can be used in each direction.

The NN, PV, and GPVE were evaluated in the present study. The ability of the optimization algorithm to reach the global maximum of MI was strongly related to the pattern of MI over the search space. A “smooth” MI pattern allows convergence to an optimal solution, while a “rough” pattern was likely to trap the optimization algorithm in local. We calculated the smoothness parameter si of the MI pattern curve by

(5) $$\text{Si}{}_{\alpha_{\text{j}}}=\frac{1}{\sum_{k=1}^{k}\left|\partial^{2}I_{\alpha_{j,k}}/\partial \alpha_{j}^{2}\right|},$$

where *α*_*j*_ represents the transformation parameters and *K* denotes the number of second derivatives present in the MI pattern curve.

#### 2.4.3. Optimization Algorithm

Even if different methods can be used to reduce the possibility of local peaks in the MI pattern, a robust optimization method is required to reach the global similarity metric maximum that corresponds to the correct spatial alignment. Different optimization algorithms can be used as a search strategy, but not all techniques are able to reach the global maximum. For instance, depending on the starting condition, local optimization methods are affected by the similarity metric pattern such that they can obtain a local solution. The use of a global optimization method in the registration process assures that the optimal solution is reached and that the correct spatial transformation parameters are defined. Some widely used global optimization techniques are the genetic algorithms (GAs). These are based on Darwin's theory of biological evolution and are implemented using stochastic information [13, 27]. The principal advantage of the GAs is that the computational complexity of the optimization algorithm does not strictly depend on the number of transformation parameters. For this reason, they can also be applied to complex registration problems, for example, the elastic registration method or global registration of multiple volumetric datasets.

In this work, we have developed a search strategy based on a multiresolution approach. Two primary steps have been defined. The first step is based on a global optimization method that uses GAs to reach a solution near the global maximum. The second step is based on a local, computationally efficient optimization method (the downhill simplex algorithm). This step originates from the solution given by the GAs and achieves the global maximum for the MI.

#### 2.4.4. Genetic Algorithm

First, the GAs require an initial definition of a population of individuals, each containing a possible solution to the problem defined in their chromosomes. In the present problem, the chromosomes included six values (*t*_*x*_, *t*_*y*_, *t*_*z*_, *r*_*x*_, *r*_*y*_, *r*_*z*_). The chromosome values for each initial solution were defined randomly in ranges that included all misalignments possible in CT-PET cardiac acquisition.

Each solution was associated with two values used to describe the new population: a fitness score corresponding to the MI value, and a reproduction probability proportional to the fitness score. Because of the high computational load of the GA method, in this step we used the NN interpolation method to calculate the MI and applied an optimal downsampling to the floating dataset. Several methods were employed to generate the new population. The first was the *elite *method used to select two or more chromosomes with the higher fitness score. The second was the *roulette wheel selection *method and was based on the calculated probabilities.

**Figure 1:**
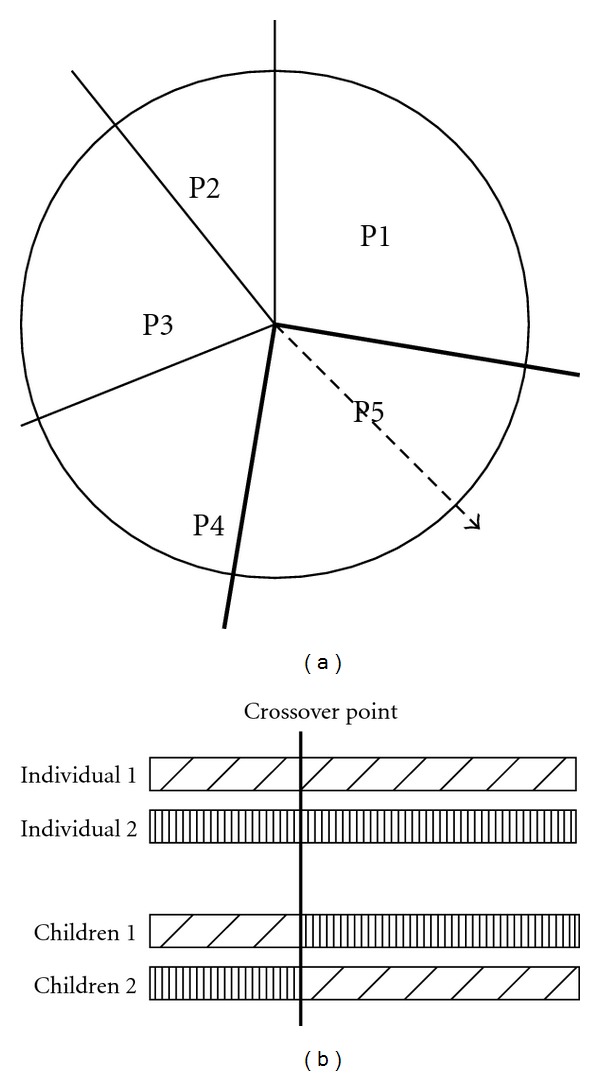
Roulette wheel selection method (a) and crossover strategy (b).

As shown in Figure 1(a), the wheel was divided into *N* sectors proportional to the probability values, and an individual was randomly extracted (arrow in the figure) to be reproduced. The process was repeated twice in order to select two individuals (individuals 1 and 2) to generate offspring via the crossover and mutation methods. The crossover (Figure 1(b)) was randomly applied to the two individuals, and their offspring were built by combining the pieces of the old chromosomes. Mutation consisted of the random replacement of some element during the process. The fitness of the new population was computed, and a new algorithm iteration was performed. The global optimization process was stopped after some number of new generations when any improvement in the best fitness value was observed. In the GA step, downsampling of the floating volume and a fast interpolation algorithm could have been used to minimize processing time.

#### 2.4.5. Downhill Simplex Method

Starting from the global solution, the local downhill simplex method was used to calculate the optimal solution. Because the main purpose of using the local method was to obtain excellent accuracy and precision within a reasonable time, the downsampled value of the floating volume was reduced in this step, and the PV interpolation method was used. The local optimization process was stopped when the maximum number of iterations was reached or when no more improvement in the best fitness value could be obtained.

## 3. Results

### 3.1. MI Metric Optimization

The MI pattern has been defined as a function of independent translational (*t*_*x*_, *t*_*y*_, and *t*_*z*_ in a range of ±30 mm) and rotational (*r*_*x*_, *r*_*y*_, *r*_*z*_ in a range of ±30°) misalignments in the synthetic models. The CT dataset has been generally used as the reference volume and the PET dataset as the floating volume. The spatial dimensions (256 × 256 × 55 pixels) and the resolution (1 mm × 1 mm × 1 mm) were the same for both models in this test. Because the interpolation artifacts in the MI pattern are most evident in the translational compared to the rotational results [24], we focused our evaluation on translational misregistration.

**Figure 2:**
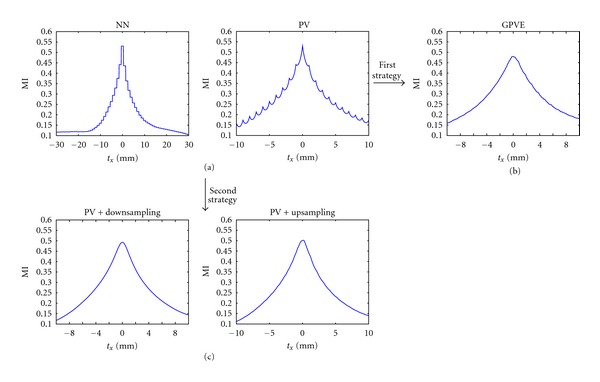
Typical MI translational pattern obtained by using different interpolation methods and strategies. Translational results for the PV method are shown in the range ±10 mm to illustrate the interpolation artifacts. Translational results for the GPVE, PV + downsampling and PV + upsampling methods are shown in the range ±10 mm for comparison of the pattern obtained against the PV curve. When the second strategy is applied, the downsampling produced a new voxel dimensions = 1.11  mm × 1.11 mm × 1.11 mm, and the upsampling a new voxel dimensions = 0.89  mm × 0.89  mm × 0.89  mm.

Figure 2(a) shows that the MI patterns depended on *t*_*x*_ when NN and PV methods were applied. Similar patterns were found for *t*_*y*_ and *t*_*z*_. According to [24], when the NN interpolation method is applied, a stairs-like pattern is obtained in translational patterns which is caused by the discrete nature of the interpolation method. Even if NN interpolation imposes a smaller computational load compared to the PV method, it does not allow intravoxel registration. The interpolation artifacts appeared also when the PV method was applied, wherever inverted arches were present in the translational curves. This occurred because the cardiac datasets we employed had the same voxel size. In fact, as described by Tsao [24], these artifacts in the MI occur when the ratio of the two datasets' spatial resolutions along a certain direction was equal either to one or was a simple rational fraction. The first is based on the GPVE method. Figure 2(b) shows the MI translational results when the GPVE method with second-order B-splines in all directions was applied. This method decreased the interpolation artifacts in the PV translational pattern. The second strategy applies a resampling of the floating dataset [28]. Figure 2(c) shows the MI translational patterns that resulted when the PV interpolation method was used and when the synthetic floating dataset was downsampled or upsampled to obtain a different resolution with respect to the reference dataset. Both downsampling and upsampling eliminated interpolation artifacts in the MI pattern. The combination of the PV interpolation method with downsampling resulted in a reduced computational load compared to the GPVE and upsampling methods. For this reason, PV interpolation with downsampling of the floating volume was used to estimate the joint histogram in the MI calculation, where the greatest downsampling factor that preserved information content of the MI was chosen.

**Figure 3:**
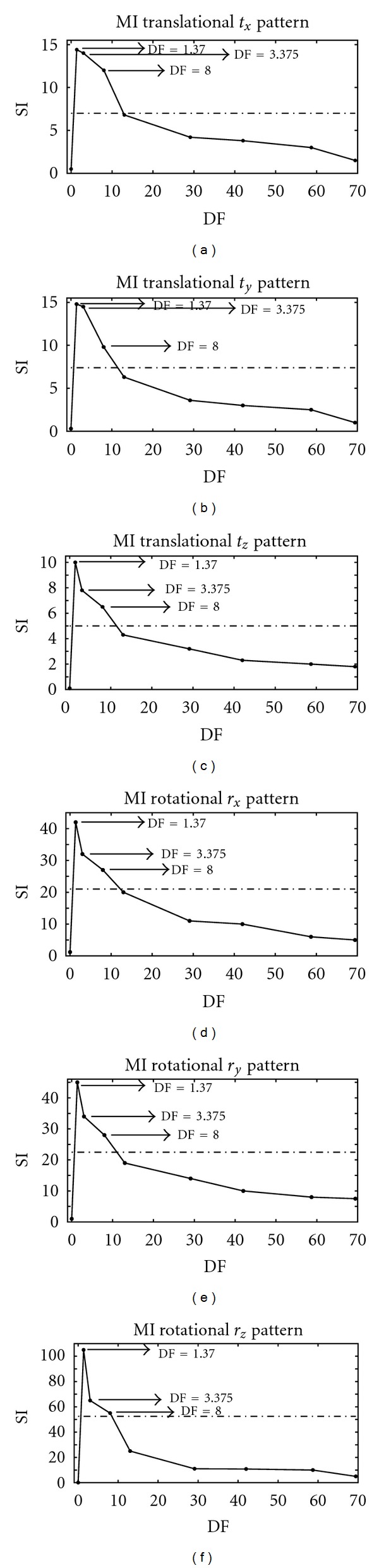
SI values for different DF values. The first row shows the SI values corresponding to the translational *t*_*x*_, *t*_*y*_, and  *t*_*z*_ MI patterns. The second row shows the SI values corresponding to the rotational *r*_*x*_, *r*_*y*_, and  *r*_*z*_ MI patterns.

To set a correct downsampling factor (DF), we also evaluated the MI patterns for each transformation parameter by applying a different DF, defined as the voxel volume measured in mm^3^ (the voxel volume of the reference dataset is 1 mm^3^), and by calculating the corresponding SI value. As shown in Figure 3, the SI value and consequently the smoothness of the MI pattern decreases when the DF value is increased. For all transformation parameters, the maximum SI value was obtained for DF = 1.37 (voxel dimensions 1.11 mm × 1.11 mm × 1.11 mm). Because higher  DF values are associated with lower computational load, we estimated the DF value by considering a minimum acceptable SI value (50% of the maximum DF value for each transformation parameter). Because of this condition, and because a multiresolution approach was used in the registration algorithm we developed, we set DF = 8 when the optimization algorithm was applied such that computational load would be lower, and DF = 3.375 when the local algorithm was applied in order to reach a higher accuracy and precision within a reasonable time.

### 3.2. PET Signal Attenuation

**Figure 4:**
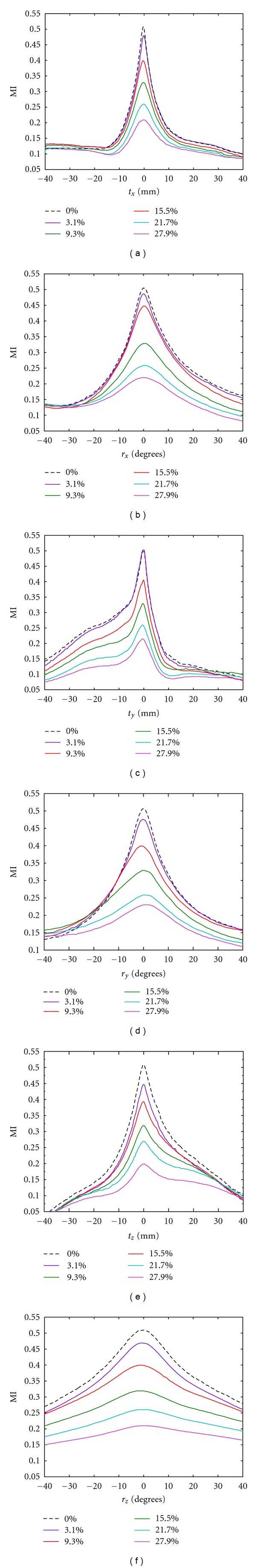
MI translational (*t*_*x*_, *t*_*y*_, and  *t*_*z*_) and rotational (*r*_*x*_, *r*_*y*_, and  *r*_*z*_) patterns for different percentages of signal attenuation in the PET model.

The PET signal in pathological conditions, as a perfusion deficit, can be missed in some regions. This aspect can affect the robustness of the registration algorithm and the MI pattern. We evaluated the influence of intensity of the PET signal on the MI pattern by varying the multiplicative kernel $\text{g}\left(\overset{®}{x}\right)\;$used in the PET model as described by Styner et al. [21]. In particular, we centered the Gaussian smoothing filter in the modeled inferior lateral wall, we set *σ* = 0.3 and we changed the maximum fading value *a*_max⁡_ to simulate different percentages of signal intensity fading (Figure 4). In particular, when the percentage is increased, the value of the MI decreases, but any variation occurs in the translational parameter values corresponding to the optimal spatial alignment. The same behavior was noticed for MI rotational patterns.

### 3.3. Definition of the Global Optimization Algorithm Parameters

The application of the GAs as the first step of the optimization algorithm allowed us to reach a solution near the global maximum. The optimal initial population size was determined by considering the accuracy of the algorithm and the computational time. We introduced a new parameter defined as

(6) $$S_{n}=e_{n}+t_{n},$$

where *e*_*n*_ represents the normalized mean error of the transformation and *t*_*n*_ represent the normalized mean execution time. We applied 30 times the registration algorithm to the cardiac models by varying the population number. We set the initial population number to 75, which corresponded to the minimum value of the *s*_*n*_ parameter.

By using an initial population number = 75, we varied the percentage of the new generation created by the elite method (*p*_*e*_) and the percentage of the remaining new population obtained by the crossover method (*p*_*c*_), and for each set of parameters, we applied 30 times the registration process to the two cardiac models. The minimum mean error was obtained for *p*_*e*_ = 0.25 and *p*_*c*_ = 0.6. The population created by the mutation operator was defined as *p*_*m*_ = (1 − *p*_*e*_) − *p*_*c*_.

### 3.4. Synthetic Data Test

The registration algorithm presented here has been evaluated by applying it 150 times to the PET and CT cardiac models, which were randomly misaligned in ranges of ±40 mm and ±20° for translational and rotational parameters, respectively. The mean error and standard deviation for each rigid transformation parameter are shown in Table 1(a). The translational mean error was less than the spatial resolution, which was set to 1 mm in each of the three spatial dimensions. The rotational mean error was less than 1.5°. The mean processing time was about 120 s.

**Figure 5:**
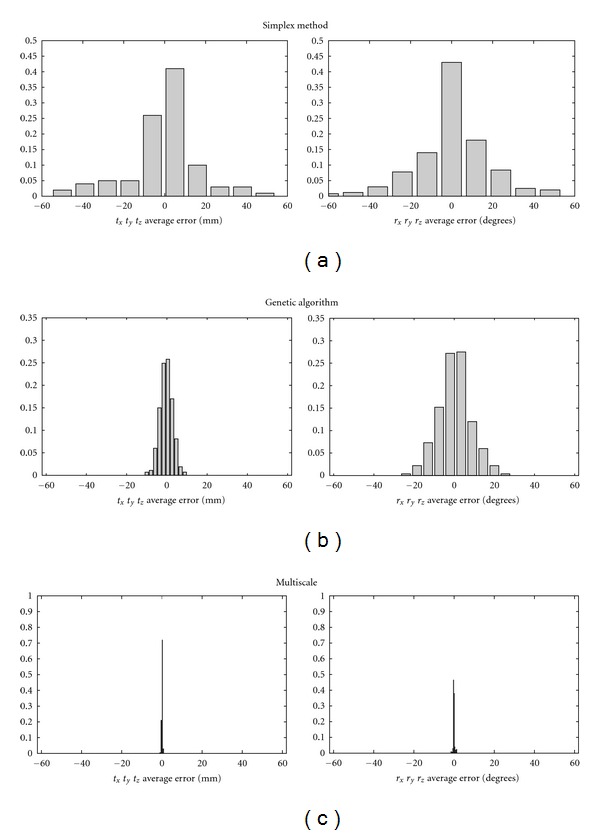
Accuracy of local, global and multiresolution optimization approaches when applied separately to the PET and CT cardiac models.

In order to also evaluate our multiresolution optimization algorithm, the local and global methods were also applied separately 150 times to the PET and CT cardiac models, starting from a random initial parameter value in the ranges previously defined. As shown in Figure 5, the simplex method had a high variability. The global genetic algorithm approach shows a lower variability although the global maximum convergence was not reached in all of the experiments. This is likely due to the imposed time constraint that limits the number of iterations in “flat fitness” state. Finally, the combination of the two methods provided a more accurate solution.

### 3.5. Real Data Test

Two real PET and CT datasets (datasets 1 and 2) were also used to evaluate the registration algorithm. First, the two datasets were manually aligned by an expert operator blinded to the results of the automatic registration, using a state-of-the-art image analysis package meant for a clinical environment (GE Healthcare, CardIQ Fusion software). These manually defined translation and rotation parameters were recorded.

The limited regions manually defined around the heart of the dataset 1 and 2 were used in the validation process to reduce the effect of the surrounding structures on the MI calculation and then on the registration algorithm. Table 1(b) shows the distribution of the transformation parameters obtained by applying our registration approach 100 times to the dataset 1 and compares them with the results from manual registration. The reported automatic value is the maximum value of the histogram of the convergence values in all 100 experiments. Standard deviation (SD) is reported if the convergence values distribution is Gaussian.

**Figure 6:**
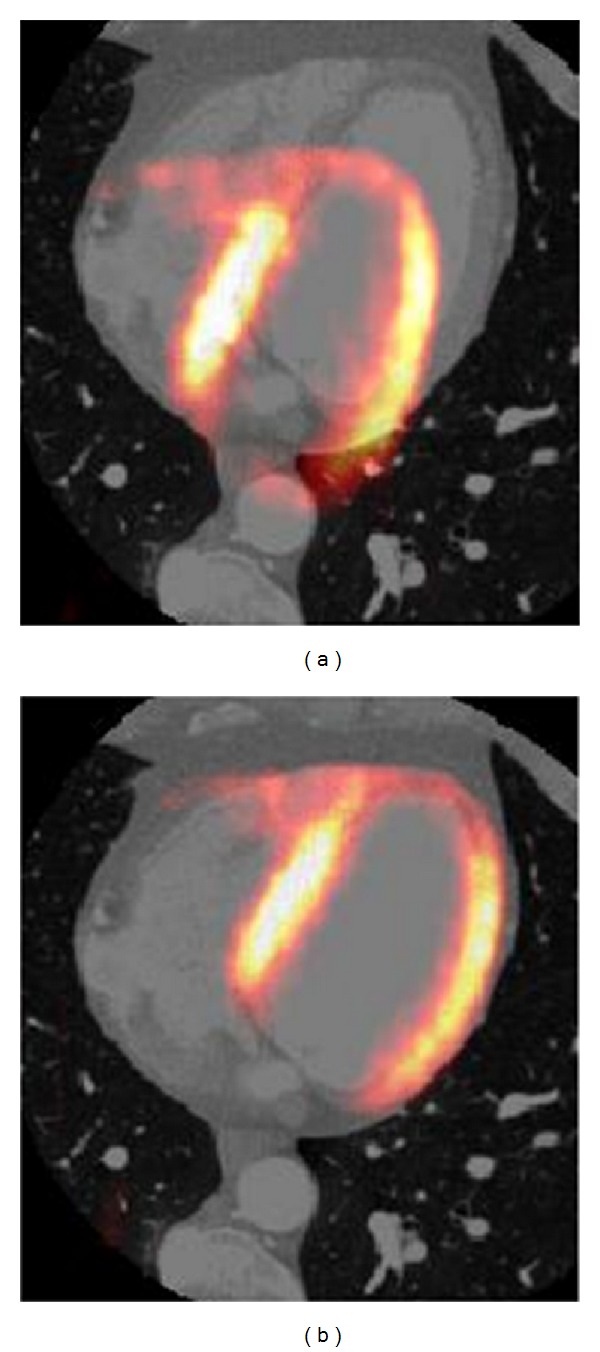
Example of misaligned (a) and aligned (b) transaxial PET and CT images corresponding to dataset 1.

For rotational parameters *r*_*x*_ and *r*_*y*_, the distribution of detected values was bimodal, with mean values *r*_*x*_ : −9.59° and −2.53°; *r*_*y*_ : 0.89° and 3.42°.  Figure 6 shows a transaxial view of the images, one from before and the other from after application of the automatic registration method.

**Figure 7:**
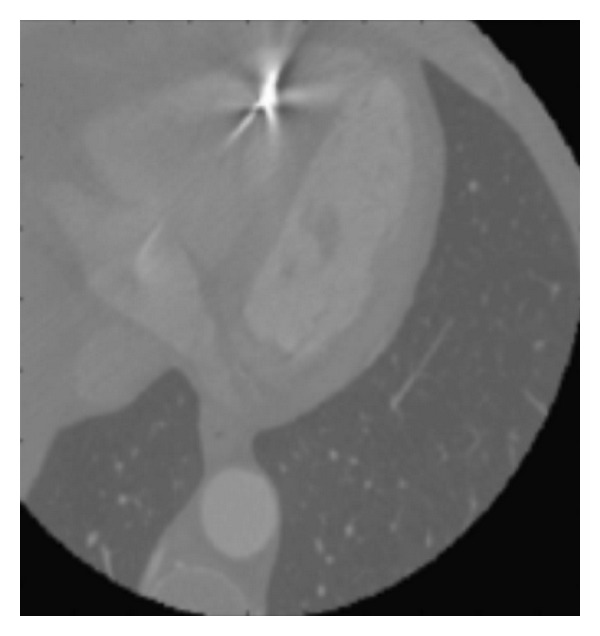
A transaxial image of the CT data volume in which an artifact caused by a metallic object is present.

**Figure 8:**
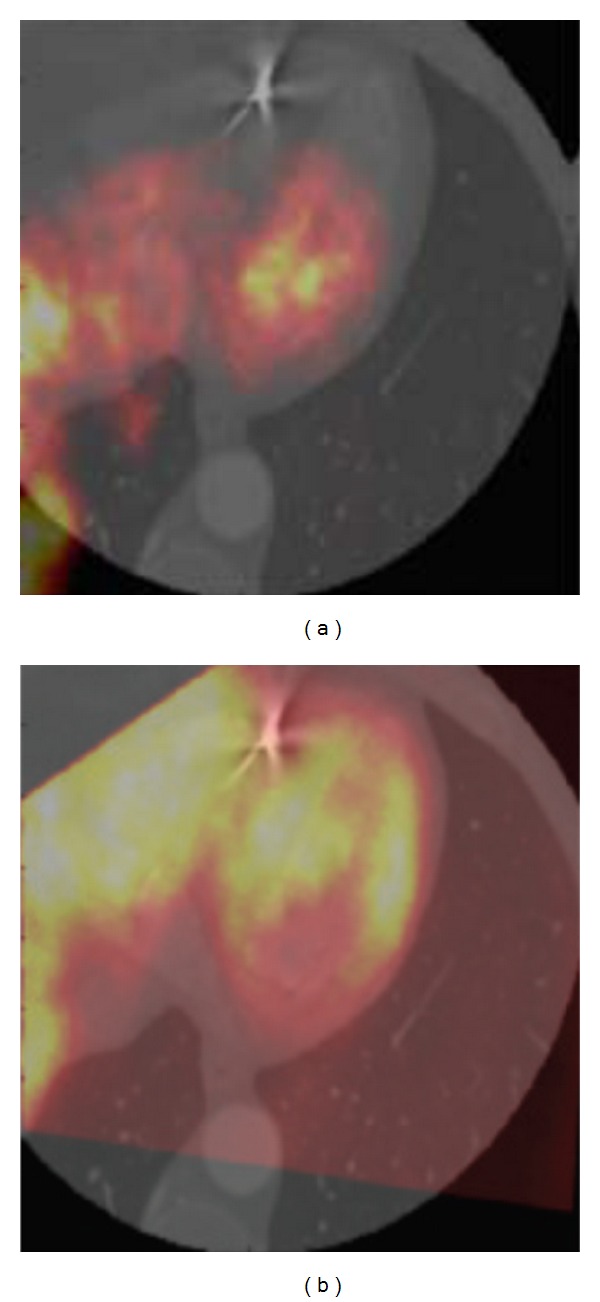
Example of misaligned (a) and aligned (b) transaxial PET and CT images in which an artifact caused by a metallic object is present.

The registration algorithm presented here was applied to dataset 2, which contained an artifact in the CT image volume caused by a metallic object (Figure 7). The behavior of the registration algorithm was similar to the one described for dataset 1. The registered PET and CT real datasets were evaluated as “well aligned” by an expert observer (Figure 8).

## 4. Discussion

Registration of PET/CT volumetric datasets is an important issue in cardiac applications in which complementary PET/CT information is utilized to find the correct diagnosis. The hardware fusion obtained by hybrid PET and CT scanners does not completely solve the misalignment problem. In the current clinical practice CT, and PET images are transferred to a proprietary workstation where the two datasets are manually registered. This task is affected by inter and intraobserver variability and requires a long processing time. Our method may replace the manual registration saving image analysis time and reducing at the same time the inter- and intraobserver variability inherent in the manual procedure. The registration algorithm is based on a rigid transformation and on the MI measurement used as the similarity metric. The optimization algorithm implemented here defines the optimal transformation that maximizes the MI value and is based on a combination of global (genetic algorithms) and local (downhill simplex) optimization methods.

MI is generally recognized as a powerful metric for registration of multimodal images, thanks to its ability to capture the similarity of datasets with different gray-lever distributions, such as in CT and PET images. However, the effectiveness of the search for the global maximum in the MI similarity function could be affected by the presence of local peaks in the MI pattern.

We have demonstrated that the MI pattern is related to the interpolation method used in the joint histogram calculation, confirming findings from earlier studies [24]. Different approaches, such as PV and GPVE interpolation, were used to solve this problem. Our choice was to implement a method that also permits a decrease in computational time for the registration process. In particular, downsampling of the floating dataset was used to “smooth” the MI pattern and was demonstrated to be equivalent to results from GPVE interpolation in this registration problem. The “smoothing” method was also evaluated by considering different percentages of intensity signal fading in the PET model that simulate a pathological defect of the myocardial wall. We determined that the method described here does not change the position of the global maximum corresponding to the optimal match in either the physiological or pathological conditions. This finding allowed us to use a multiresolution registration approach. Fast interpolation with a high downsampling factor was utilized in the first stage of the registration process, and a global GA optimization method was implemented. The global optimization method integrated into the registration process was also assessed, and optimal parameters to describe the genetic algorithms were defined as minimizing the computational time and maximizing accuracy of the solution. When convergence to a local maximum was reached, an accurate, local optimization method performed the final registration, using the full data resolution and accurate interpolation algorithm. We demonstrated that the combined multiresolution optimization method yielded higher accuracy compared to results from either the global or local methods. Application of the registration algorithm to the synthetic dataset confirmed that the automatic algorithm implemented allowed us to reach the correct spatial alignment of the modeled PET/CT datasets. The results obtained for the real dataset enforces the possibility to apply the developed registration algorithm to real datasets, even when image artifacts were present. Development and assessment of the registration process were performed by two synthetic PET and CT cardiac models used to simulate real registered datasets. The developed models do not take in account any attenuation correlation between the PET and CT, they do not simulate the cardiac and breathing cycle. These issues may be included in a more sophisticate models. Moreover, during the real datasets test, the convergence of two rotation parameters (i.e., *r*_*x*_ and *r*_*y*_) was not robust as expected, reaching a local maximum of corresponding MI value. This can be due to the circular-like shape of the left ventricle in PET data in the *x*-*y* plane or to the presence of surrounding structures not modeled in synthetic datasets. This issue merits further exploration and validation to improve the robustness of the algorithm.

A possible limitation of the proposed approach is the use of a rigid transformation, that cannot take into account either the complex nonrigid motion of the heart during the cardiac cycle or the various image distortions present in the CT and PET acquisition processes. However, rigid registration is commonly used in clinical practice and is considered to be acceptable for reaching the correct diagnosis, particularly when electrocardiographic gating is not used in PET imaging to save acquisition time. In this case, the borders of the left ventricle in PET images appear to have been too “smoothed” to obtain a voxel-to-voxel registration. Our expectation is that a nonrigid registration procedure can be inserted into the local optimization step of the proposed algorithm, via one of the several approaches available.

## 5. Conclusion

We have demonstrated that cardiac PET/CT image registration can be effectively and automatically performed using a two-step approach. The first step consists of identification of a global maximum of mutual information by using a genetic algorithm which in turn utilizes downsampled data and a fast interpolation algorithm. The second step completes the registration with a high-resolution local optimization algorithm. The method proposed here may improve the effectiveness of hybrid PET/CT scanners for use in the joint assessment of CAD and myocardial perfusion in cardiac disease.

## Figures and Tables

**Table 1:** Accuracy of the registration algorithm applied to the synthetic (a) and real (b) datasets. When the real dataset is considered, the results of the manual registration are also shown. The manual registration has been performed by an expert operator blinded to the results of the automatic registration and used as reference for the error computation.

			*t_x_* (mm)	*t_y _*(mm)	*t_y _*(mm)	*r*_*x*_ (°)	*r_y_* (°)	*r_z_* (°)
(a) Synthetic								
	Automatic	Mean Error	0.083	0.054	0.037	0.117	0.286	0.201
		standard deviation (SD)	0.108	0.089	0.097	0.215	0.2718	0.397
(b) Real								
	Manual		−25.61	18.51	−4.9	−9.45	0.86	−2.01
	Automatic	Mean error	−25.89	18.485	−5.157	−9.59	0.89	−2.29
		standard deviation (SD)	0.238	0.146	0.236	—	—	0.825
